# The current state and future of T-cell exhaustion research

**DOI:** 10.1093/oxfimm/iqad006

**Published:** 2023-07-08

**Authors:** Edward Jenkins, Toby Whitehead, Martin Fellermeyer, Simon J Davis, Sumana Sharma

**Affiliations:** Kennedy Institute of Rheumatology, University of Oxford, Oxford OX3 7FY, UK; Medical Research Council Human Immunology Unit, John Radcliffe Hospital, University of Oxford, Oxford OX3 9DS, UK; Medical Research Council Human Immunology Unit, John Radcliffe Hospital, University of Oxford, Oxford OX3 9DS, UK; Medical Research Council Human Immunology Unit, John Radcliffe Hospital, University of Oxford, Oxford OX3 9DS, UK; Medical Research Council Human Immunology Unit, John Radcliffe Hospital, University of Oxford, Oxford OX3 9DS, UK

**Keywords:** T-cell activation, T-cell exhaustion, immunotherapy, inhibitory receptors, in vitro exhaustion

## Abstract

‘Exhaustion’ is a term used to describe a state of native and redirected T-cell hypo-responsiveness resulting from persistent antigen exposure during chronic viral infections or cancer. Although a well-established phenotype across mice and humans, exhaustion at the molecular level remains poorly defined and inconsistent across the literature. This is, in part, due to an overreliance on surface receptors to define these cells and explain exhaustive behaviours, an incomplete understanding of how exhaustion arises, and a lack of clarity over whether exhaustion is the same across contexts, e.g. chronic viral infections versus cancer. With the development of systems-based genetic approaches such as single-cell RNA-seq and CRISPR screens applied to *in vivo* data, we are moving closer to a consensus view of exhaustion, although understanding how it arises remains challenging given the difficulty in manipulating the *in vivo* setting. Accordingly, producing and studying exhausted T-cells *ex vivo* are burgeoning, allowing experiments to be conducted at scale up and with high throughput. Here, we first review what is currently known about T-cell exhaustion and how it’s being studied. We then discuss how improvements in their method of isolation/production and examining the impact of different microenvironmental signals and cell interactions have now become an active area of research. Finally, we discuss what the future holds for the analysis of this physiological condition and, given the diversity of ways in which exhausted cells are now being generated, propose the adoption of a unified approach to clearly defining exhaustion using a set of metabolic-, epigenetic-, transcriptional-, and activation-based phenotypic markers, that we call ‘M.E.T.A’.

## Introduction

The exhaustion of cytotoxic T-cells was originally described in the context of chronic lymphocytic choriomeningitis virus (LCMV) infections of mice in which the depletion of functional effector cells was noted [1]. Several years later it was shown that these cells were not depleted but were instead present but dysfunctional [2]. The process of how effector T-cells become dysfunctional has been a topic of interest for many years, whose study has especially benefited from advancements in the fields of single-cell transcriptomics and T-cell receptor (TCR) sequencing. These methods allow mapping of the transcriptomic profile of a heterogeneous cell population in combination with the identification of antigen-specific T cells, without relying on functional readouts. With the help of these new approaches, it is now known that exhausted T cells comprise distinct immune cell types of a specific lineage that differ from effector or memory T cells and play a central role in cancer, autoimmunity and chronic infections.

In its simplest sense, T-cell exhaustion refers to a dysfunctional state of the T cell that is characterized by an impaired ability to respond to infected cells or tumours. In a chronic infection state, the lingering presence of an invading pathogen leads to prolonged antigen stimulation resulting in T-cell dysfunction. In a tumour setting, T cells become exhausted due to chronic antigen exposure leading to reduced effector function [loss of interleukin-2 (IL-2), tumour necrosis factor alpha (TNF-α) and Interferon-gamma (IFN-γ) production]. Accordingly, CD8^+^ cells can infiltrate the tumour microenvironment (TME) but are then unable to mount an effective anti-tumour response, allowing continued growth of the tumour. The exhausted state of a T cell is driven by a unique transcriptional programme that generates a specific phenotypic state.

Much of the current interest in T-cell exhaustion resulted from the discovery that it imposes major constraints on the therapeutic efficacy of chimeric antigen receptor (CAR) T cells, prompting attempts to create exhaustion-resistant or -reversible CAR T-cell platforms (see, e.g. Refs [3–5] as an example of the work of just one laboratory). However, how exhausted T cells emerge has been a field of active study for many years. Early studies on T-cell exhaustion suggested that previously functional T cells terminally differentiate to become exhausted T cells. But with the emergence of single-cell techniques and the ability to characterize the cell populations based on transcriptomic profiles, this model is having to be revised. A T-cell exhaustion programme is now thought to occur alongside the normal differentiation of T cells [6]. The notion is also that cells at any stage of differentiation can initiate the exhaustion programme. The way exhausted cells are defined is also changing and it is thought that among ‘exhausted’ T cells, there exists a subset of precursors that proliferate and constantly replenish a pool of terminally differentiated cells that are proliferation-incompetent and are sometimes referred to as ‘truly’ exhausted T cells [7, 8].

The different types of exhausted T cells, their biology, developmental paths and their transcriptional and epigenetic features have been comprehensively reviewed previously [9, 10]. Here, we consider how exhausted T cells can be defined based on transcription factor activity, expression of inhibitory receptors, effector functions, metabolic status and epigenetic status. We also discuss the new *ex vivo* and *in vitro* approaches for generating exhausted T cells and how these definitions are being used to identify these cells. For the purposes of this review, we will focus mainly on the biology of CD8^+^ T cells.

## The different definitions of exhausted T cells

### Transcriptomic properties/states of exhausted T cells

Understanding their transcriptional programmes is key to the identification of the cellular processes leading to the differentiation of any cell type. Studies that characterize cell-type-specific gene-regulatory networks in exhausted T cells have been crucial in this regard and a clear conclusion from these studies is that these cells have a transcriptional profile which is different from that of memory or effector CD8^+^ T cells, not only in terms of the differential expression of transcription factors (TFs), but also in the way the expressed transcription factors in turn regulate the underlying gene-regulatory networks. Using transcription factor expression profiles is a commonly deployed approach to define the exhaustion state. More comprehensive studies combine the expression profiles and gene-regulatory networks driven by the transcription factors, including TOX, nuclear receptor subfamily 4 group A (NR4A), T-bet, EOMES, nuclear factor of activated T-cells (NFAT) and other NFAT-driven and TCR-responsive transcription factors (such as IRF4 and BATF) to characterize exhaustion states [11].

The NFAT family of transcription factors comprises a class of very well-studied proteins in relation to T-cell activation, T-cell anergy and T-cell exhaustion. T cells express three of the four calcium-regulated NFAT proteins: NFAT1, NFAT2 and NFAT4. The functions of NFAT transcription factors depend on the T-cell differentiation state. In the setting of activation, NFAT proteins work coordinately with activator protein 1 (AP1) transcription factors, forming heterodimers that promote the transactivation of many cytokine genes [12]. In the context of chronic LCMV infection, the expression balance of NFAT and AP-1 shifts towards high NFAT expression, leading to the formation of NFAT homodimers. NFAT homodimers, on the other hand, bind to the promoter regions of a number of genes encoding inhibitory receptors, such as *PDCD1*, *LAG-3* and *HAVCR2* (the gene encoding TIM-3) [13, 14]. In addition to NFAT, other transcription factors, such as IRF4 [15], BATF [16] and NR4A1 [17], which function downstream of TCR signalling, also contribute to the formation of the exhausted state. These transcription factors are mostly known to be involved in the expression of multiple inhibitory receptors including PD-1, in this way constraining effector function.

Transcription factors such as T-bet and EOMES have very distinct roles in effector and memory cells compared with exhausted cells. T-bet drives the expression of effector molecules such as IFN-γ in effector cells whereas EOMES is important for memory formation. In effector cells, T-bet also represses the expression of PD-1 [18]. In chronic viral infections, the expression of T-bet is reduced in virus-specific CD8^+^ T cells, with the level of reduction correlating with the degree of T-cell effector dysfunction. EOMES expression, on the other hand, is upregulated in exhausted CD8^+^ T cells during chronic infection [19]. In addition to expression differences, the gene-regulatory networks controlled by EOMES are highly context-specific and the exhausted-cell network overlaps very little with that of effector or memory cells [20]. Recent work suggests that the relative level of T-bet and EOMES in T-cells could define exhaustion since, in LCMV infections, exhausted cells had a higher ratio of nuclear EOMES: T-bet than memory T cells [21].

Another transcription factor with a key role in T-cell exhaustion is TCF-1, which is encoded by *TCF7.* TCF-1 has been previously implicated in the production and maintenance of memory CD8^+^ cells and has been suggested to have a similar role in the maintenance of stem-like pre-exhausted cells [8]. Chen *et al.* [22] used single-cell RNA-seq and lineage tracking in the LCMV-infection mouse model of exhaustion and found that the separation of effector from exhausted CD8^+^ T-cell lineages is mediated by TCF-1 early during chronic infection. The exhaustion lineage in this model was marked by expression of PD-1 and TCF-1 and characterized by low expression of Gzmb and Klrg1, whereas the effectors included cells expressing terminal differentiation markers such as TIM-3 and CD39. In the exhaustion cells, *TCF7* might also coordinate transcriptional networks involving EOMES, c-Myb, and Bcl-2, which are known to reinforce exhaustion and persistence.

One of the newest transcription factors implicated in exhaustion is the Thymocyte selection-associated HMG Box (TOX) transcription factor. Initial network analysis identified TOX as one of the most differentially connected TFs expressed by memory and exhausted T cells, suggesting a possible important role in exhaustion [20]. This was later clarified by work showing that TOX could be the master regulator driving an exhaustion phenotype marked by the upregulation of inhibitory receptors, decreased cytokine production and epigenetic remodelling in chronic infection or in a tumour setting [23–27]. TOX is required for the formation of exhausted CD8^+^ T cells via chromatin remodelling and alteration of gene expression profiles. Additionally, TOX promotes the expression of genes that are often associated with exhaustion, including the expression of inhibitory receptors such as PD-1, TIM-3 and LAG-3 and transcription factors EOMES, TCF-1 and CD38. TOX expression is regulated by NFAT, a key regulator of T-cell exhaustion and dysfunction (discussed above).

While the expression profiles and gene-regulatory networks of these transcription factors have been well studied in the context of exhaustion (summarized in Table 1), how these expression patterns ought to be used to define exhaustion still needs clarification. It has been known for a long time now that a spectrum of exhausted T cells will exist, from naïve, undifferentiated cells to terminal differentiation cells. The expression of T-bet and EOMES was originally used to define subpopulations within the exhausted cells. A population of proliferation-competent progenitors was defined as Tbet^hi^Eomes^lo^ and terminal effectors as Tbet^lo^Eomes^hi^ [19]. Recent work from Beltra *et al.* [28] has fine-tuned the transcription factor-based definition of exhaustion states. Now, exhaustion is defined as a spectrum of phenotypes with four developmental subsets forming depending on the state of the TME and/or peripheral tissue. These subsets consist of (1) TEX^prog1^, which are quiescent lymphoid-tissue resident non-proliferative progenitor cells, have high TCF-1 expression, are slowly dividing and have good persistence; (2) TEX^prog2^, which are TCF1+, highly proliferative, activated progenitor cells with good persistence; (3) TEX^int^ comprising an intermediate population with some effector activity and migratory capacity, but no TCF-1 expression and (4) a terminal population, TEX^term^, that is more cytotoxic but non-recirculating. This scheme also clarified how the other transcription factors change in the four developmental stages of exhaustion. For example, T-bet is a major regulator of the TEX^int^ population, with TOX expression negatively correlated with T-bet expression. TOX expression decreased as T-bet expression increased and cells progressed from progenitor cells to intermediate cells. TOX expression, however, increased in terminal cells, leading to a decrease in the expression of T-bet. EOMES, on the other hand, was expressed by the TEX^prog1^ but downregulated in the TEX^prog2^ and TEX^int^ subsets. EOMES, however, rebounded back to high expression in the TEX^term^ subset [28]. The levels of expression of transcription factors are therefore helpful for defining distinct states of exhaustion.

**Table 1.** Summary of transcription factors expression associated with effector, progenitor and terminally exhausted T cells

Transcription factor	Effector T cell	Progenitor exhausted T cell	Terminally exhausted T cell	References
TCF-1	High	High	Low	[8, 22]
Ratio of T-bet and EOMES	More T-bet	More T-bet	More EOMES	[18, 19, 21]
BATF^a^	Complexed with IRF4 promotes effector function	High	High	[11, 15, 29, 30, 31, 32]
NFAT	Heterodimer with AP-1	Unclear	NFAT homodimer	[14]
PRDM1	High	Low	High	[30, 33]
NR4A	Low	High	High	[17, 24]
TOX	Low	High	High	[23, 25, 26, 27]

a BATF is highly expressed in terminally exhausted T cells [15]; however, recent papers show that with conjugation with IRF4, it has the capacity to divert the cells away from exhaustion-like programmes [31, 32].

### Sustained expression of inhibitory receptors

The identification of inhibitory receptors, including PD-1, CTLA-4 and TIM-3, as ‘exhaustion markers’ was one of the first key definitions of T-cell exhaustion [34]. It is thought that in the continuous presence of antigens, inhibitory receptors affect exhaustion by dampening the activation of antigen-specific T cells. PD-1 is the most-studied molecule in the context of exhaustion. Normally in T cells, PD-1 expression increases in response to TCR stimulation. In the context of exhaustion, however, there is constitutive PD-1 expression and PD-L1 engagement, inhibiting CD8^+^ T-cell function. Reagents that block the PD-1/PD-L1 interaction, e.g. anti-PD-1 and anti-PD-L1 antibodies, induce an anti-tumour response via the reinvigoration and expansion of CD8^+^ cell populations and by increasing their cytotoxic activity [35]. In addition to PD-1, in a chronic infection with viruses, antigen-specific CD8^+^ T cells in both animals and humans will co-express other inhibitory receptors including LAG-3, CD244 (2B4), CD160, TIM-3 and CTLA-4 [34]. In the LCMV model, exhausted T cells expressed a number of these inhibitory receptors and the severity of the infection correlated with both the levels and the number of different inhibitory receptors expressed per cell [36]. SLAMF6 has also been identified as a marker of ‘progenitor exhausted’ cells, and TIM-3 as a marker on ‘terminally exhausted’ cells, whereas PD-1 is found on both subsets, with increased expression on the terminally exhausted cells [33]. Expression of inhibitory receptors on exhausted T cells is attributed to the master regulator of exhaustion TOX, which increases chromatin accessibility at the promoters of these genes. Importantly, TOX is required for the continuous PD-1 expression by reducing its degradation and promoting its recycling back to the cell surface [37]. Expression of inhibitory receptors in exhaustion is believed to occur for all lymphocytes, with NK cells and B cells also upregulating PD-1 in their exhausted states. Other receptors unique to each cell type are also present, such as KIRs in NK cells and CD11c in B cells (reviewed in detail by Roe [38]).

The functions of inhibitory receptors in exhausted T cells are poorly understood. These receptors are not exclusively found on exhausted cells, with various receptors playing different physiological roles during canonical T-cell activation. In the ‘tide model’ of acute T-cell activation [39], inhibitory receptors, including PD-1 and CTLA-4, are upregulated in order to counteract stimulation through the TCR and co-stimulatory receptors such as CD28, preventing excessive responses. It could be argued that this explains the pattern of inhibitory receptor expression in chronically stimulated exhausted T cells. The extra stimulation occurring in disease contexts may take the process too far, with increased inhibitory receptor signalling resulting in the dysfunction of exhausted T cells. Although PD-1 is not required for exhaustion to be established in LCMV-infected mice, its absence produces more severe exhaustion [40]. This indicates that PD-1 may still be functioning in its canonical role of preventing over-stimulation of cells even in exhausted cells. Alternatively, expression of the inhibitory receptors could merely reflect underlying transcriptional and epigenetic changes and have no exhaustion-specific function. This is supported by the fact that PD-1 blockade appears to have a minimal effect on the TIM-3+PD-1+ terminally exhausted-cell population, indicating that PD-1 is not actively inhibiting the cytotoxic function or proliferation of these cells [33], although the presence of other co-inhibitory receptors, such as LAG-3 and TIGIT, may restrict the reinvigoration effect of only targeting PD-1. Co-blockade of both LAG-3 and PD-1 has a synergistic effect on LCMV-induced exhaustion, enabling greater T-cell function and clearance of virus [36]. Thus, inhibitory signalling networks from multiple inhibitory receptors acting non-redundantly may be resulting in T-cell exhaustion and dysfunction, and could be limiting the effects of targeting only one receptor in checkpoint blockade. Ultimately, the role of inhibitory receptors in exhausted T cells is an area which obviously requires more investigation, to determine whether inhibitory receptors expressed on exhausted cells are functional or simply a by-product of underlying transcriptional changes.

### Loss of effector function

The early studies mainly defined exhaustion as the inability of cells to produce effector molecules. More than two decades ago, in a seminal study, Zajac *et al.* [2] identified T cells in chronic viral infections that persist but do not produce IFN-γ. Subsequently it was suggested that as T cells become exhausted they sequentially reduce the production of IL-2 and then TNF, followed by the complete loss of IFN-γ [41–43]. More recently, it has become clear that the way exhausted T cells lose their effector function is not completely hierarchical. In fact, exhausted T cells should not be viewed as cells that are devoid of effector function—they can produce inflammatory cytokines and granzymes and are important for maintaining some control over chronic infections and tumours. The heterogeneity of exhausted cells comes into play here once again. In the four-stage hierarchical developmental pathway model of Beltra *et al.*, it became clear that precursor exhausted cells that express PD-1 have reduced effector molecule expression but the intermediate population (TCF-1^−^/T-bet^high^) downregulates PD-1 expression, which coincides with their regaining the ability to proliferate and produce effector molecules. The expression of TCF-1 is thought to be important here as it promotes the expression of an array of key effector function-associated transcription regulators, including Foxo1, Zeb2, Id3 and EOMES [44]. In addition, exhausted CD8^+^ T cells are also poor responders to homeostatic cytokines such as IL-7 and IL-15, although this feature is rarely used to define exhausted T cells [45].

### Altered metabolic activity

In the process of becoming exhausted, T cells alter their metabolic activity. In the early stages, glycolysis is down-regulated, which is attributed to the expression of inhibitory receptors [46]. This has been studied mainly in the context of PD-1 signalling and it is known that the cytosolic tail of PD-1, which contains an immunotyrosine inhibitory motif and an immunotyrosine switch motif, recruits the phosphatase SHP-2, which antagonizes signal transduction by the TCR. One pathway affected is signalling through the PI3K/AKT/mTORC1 axis which, when reduced, blocks glucose uptake by downregulating Glut1 expression, and glycolysis by suppressing the expression of the key glycolytic enzyme, hexokinase 2 [47, 48]. In the absence of glycolysis, it is proposed that exhausted T cells rely on fatty acid oxidation [46]. Exhausted tumour infiltrating T cells also have a marked loss of mitochondrial mass and function [46, 49]. This is mainly caused by loss of PPAR-gamma co-activator 1α (PGC1α), which is responsible for mitochondrial biogenesis [50]. PGC1α is repressed by the transcription factor Blimp-1 which is active under continuous TCR stimulation and hypoxic conditions [51]. Overall, the metabolic alterations of exhausted T cells are yet to be exhaustively characterized and for now, the known changes are not used to define exhaustion. It could well be that the changes are wholly secondary to the sustained expression of inhibitory receptors.

### The epigenetic status of exhausted T cells

Studies in murine chronic infection and tumour models have shown that epigenetic rewiring is a key part of the exhaustion differentiation programme, underlying the archetypal transcriptional network changes discussed above. It is the extensive epigenetic changes or ‘scars’ that exhausted cells carry which leaves them unable to acquire functional potential even after the antigen is eliminated. Antigen-specific exhausted T cells in the LCMV model were found to have a net increase in chromatin accessibility, particularly at enhancer sites for exhaustion-associated genes, including *Havcr2* and *Pdcd1* [52]. These changes were specific for chronically stimulated cells, with unique epigenetic alterations producing ∼6000 differentially accessible genetic loci compared with acutely stimulated cells [30, 53, 54], further reinforcing the differences in the differentiation of exhausted versus effector or memory cells. TOX had a key role in this reprogramming, with removal of TOX increasing chromatin accessibility at effector-related genes in LCMV-specific T cells, via the recruitment of a variety of chromatin remodelling proteins [23]. *De novo* DNA methylation of effector genes by Dnmt3a, in particular, is implicated as a regulator of exhaustion, with new DNA methylation at *Tcf7*, *Ifng* and *Myc* loci being linked to the differentiation of the exhausted cells: deletion of *Dnmt3a* resulted in reduced cell dysfunction and heightened proliferation [55].

Epigenetic differences between the stem-like and terminally exhausted subsets in LCMV-specific murine T cells have also been identified. Stem-like cells had chromatin accessibility regions (ChARs) at memory-associated genes (*Il2*, *Tcf7*) and co-stimulatory receptors (*Icos*, *Cd28*), whereas for the terminally exhausted subset, inhibitory receptors (*Pdcd1*, *Havcr2*) and cytotoxic effector genes (*Gzmb*) had increased chromatin accessibility [23, 30]. Similar differences were identified in human tumour-specific exhausted cells, suggesting a conserved epigenetic programme exists across exhausted cells [33]. Crucially, these alterations in chromatin accessibility were reflected in the transcriptomes of these cells [23, 30, 33], linking chromatin remodelling with the phenotypic changes discussed above.

There is great therapeutic interest in understanding the epigenetic plasticity of exhausted T cells as the unique epigenetic reprogramming of exhausted T cells may present a barrier to the reinvigoration of T cells by immune checkpoint blockade (see review by Dolina *et al.* [56] for more on reversal of T-cell exhaustion). ATAC-seq of exhausted murine tumour-specific T cells following anti-PD-1 treatment exhibited only a small change in ChAR distribution relative to non-treated cells, with limited changes in the associated transcriptional networks [54]. Work in LCMV mouse models indicated that there is an irreversible epigenetic transition following initial reversible transitions in the exhaustion spectrum suggesting that, beyond a certain point, cells cannot be fully restored to an effector phenotype [53]. However, inhibition of DNA methylation or use of DNA demethylating agents in exhausted cells prior to PD-1 blockade boosted the reinvigoration of exhausted cells, indicated by the increased proliferation and effector function of LCMV-specific T cells [55]. Although immune checkpoint blockade by itself may not overcome the epigenetic barrier presented by exhausted T cells, targeting chromatin remodelling could improve the therapeutic outcome of checkpoint blockade. Recent work by Ford *et al.* [57] shows that even in terminally exhausted cells, large portions of DNA contain open structures but gene expression from these regions require strong co-stimulation. Additionally, the hypoxic environment of the TME further contributed to the impaired gene expression. Therefore, restoring oxygen availability or improving co-stimulation could be an alternative way to help reinvigorating exhausted cells that have epigenome markers that are indicative of terminal exhaustion [57].

Although not commonly used as a marker of exhaustion, the epigenetic programmes underlying the transcriptional and phenotypic changes observed are unique to this differentiation pathway. While whole epigenomes are heterogeneous across exhausted-cell populations, epigenetic events including greater chromatin accessibility and *de novo* DNA methylation events at specific loci, such as *Pdcd1*, are likely to be useful for distinguishing exhausted cells from other T-cell populations.

**Figure 1.**
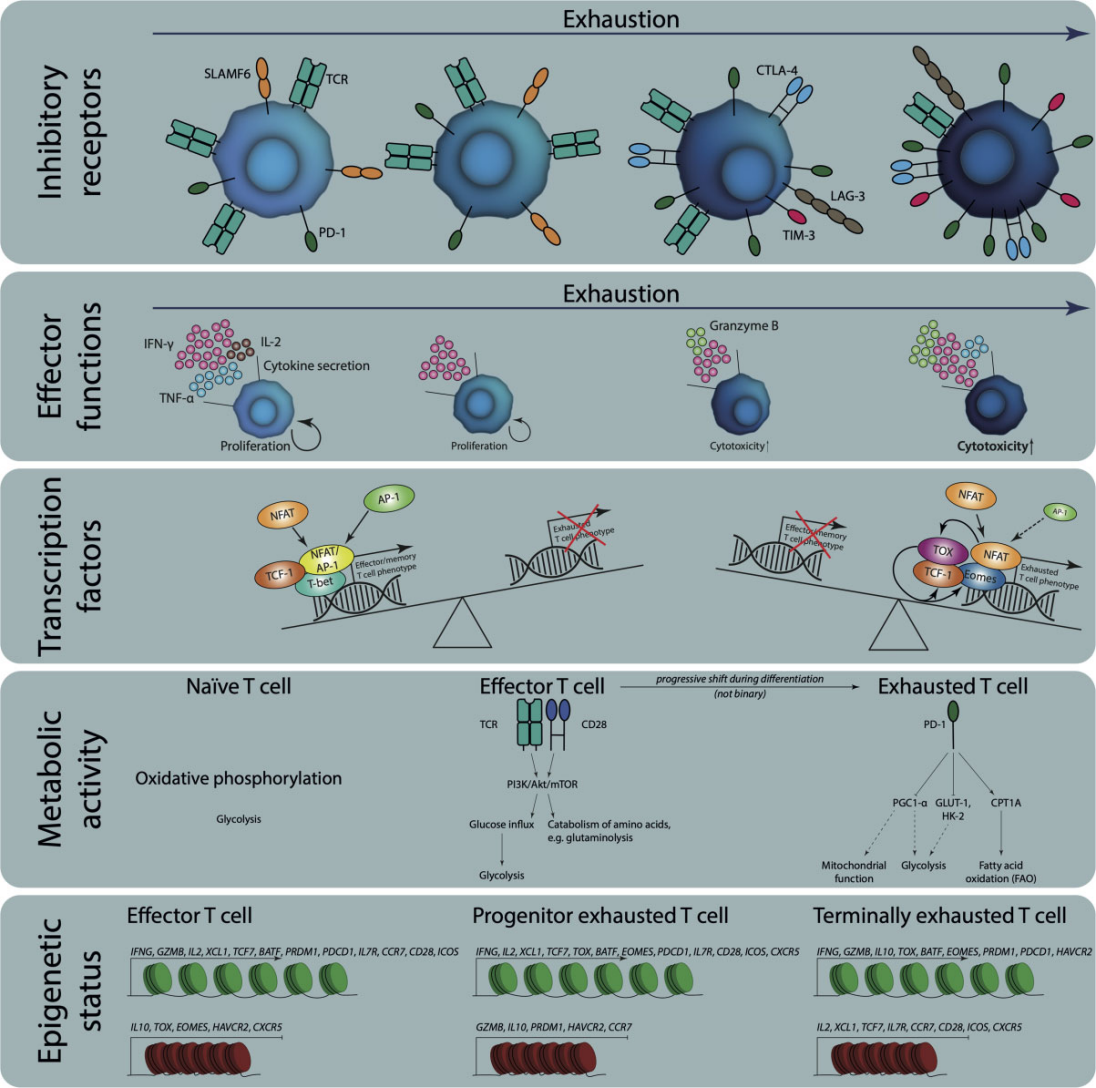
The most common properties used to define exhausted T cells. The expression of inhibitory receptors, their effector functions, expression of specific transcription factors, metabolic activity and their epigenetic status are used to define exhaustion of T cells.

To summarize, most studies use the inhibitory receptor expression, effector functions and, less commonly, transcriptional states to define the exhausted T-cell phenotype (summarized in Fig. 1). The expression of inhibitory receptors is the most used definition, as it is closely tied to other features of exhaustion (e.g. the epigenetic and metabolic states) [58]. However, these molecules are upregulated during the normal course of T-cell stimulation, and so inhibitory receptor-based definitions of exhausted T cells will be unreliable, and we argue that this alone cannot be used to adequately define cells in this state. Effector functions are also commonly used but the effector behaviour of exhausted cells is indistinguishable from that of anergic cells. The expression of transcription factors can help to define the exhausted state, with the expression of TOX being the most helpful marker. Given these difficulties and the developmental heterogeneity of exhausted cells, great care must be exercised in identifying these important cells, especially when working with T cells generated *in vitro*, which we will now discuss in detail.

## Approaches to study/generate exhausted T cells

### Isolation of exhausted cells from mouse models and human tissue

*In vivo*/*ex vivo* approaches have yielded fundamental insights into the requirements for the induction and progression of T-cell exhaustion (i.e. persistent antigen exposure).

*In vivo* mouse models are most employed to study T-cell exhaustion/hyporesponsiveness. Exhaustion was first characterized, and continues to be studied, by infecting mice with either LCMV clone 13 or the LCMV-D (DOCILE) strains, which both establish a persistent infection at a high titre [1, 2, 15, 18, 20, 23, 36, 42, 59, 60–64]. Experiments will typically involve analysing the function and phenotype of adoptively transferred transgenic antigen-specific T cells (e.g. LCMV P14) taken from the spleen/lymph node/tissue of infected mice at different time points. Caveats of this approach are that it takes a substantial time commitment to produce exhausted cells in mice (∼30 days; [65]) and yields limited numbers of cells for experimentation. To illustrate this, one study using the LCMV challenge model to isolate exhausted T cells reports using 10 mice for a single scRNA-seq experiment [66]. Despite this, mouse models provide the flexibility of performing perturbation experiments to characterize gene functions but the translation from mouse to humans remains, of course, a challenge in many cases.

Isolating exhausted cells directly from human tissue is also a commonly used approach. Exhausted T cells in humans were first identified and studied from patients with chronic viral infections [i.e. human immunodeficiency virus-1 (HIV), hepatitis B virus (HBV), hepatitis C virus (HCV) [16, 67–77]]. Around the same time, exhausted tumour infiltrating T cells (defined at the time by inhibitory receptor upregulation and reduced cytokine production when stimulated *ex vivo*) were isolated, and continue to be identified, from a variety of tumours (e.g. human metastatic melanoma tissue, ovarian cancer, non-small cell carcinoma, hepatocellular carcinoma, Hodgkin lymphoma, chronic lymphocytic leukaemia and chronic myeloid leukaemia [78–93]), subsequently leading researchers to establish several mouse tumour models to study exhaustion [65, 90, 94, 95]. Studying human tissue relies on obtaining patient blood from virus-infected individuals or tumour explants followed by isolation of immune cells using a Ficoll-Paque gradient and subsequent experimentation with known viral or tumour antigen-specific T cells (further isolated or stained via MHC tetramers) [84]. For tissue, this requires performing mechanical dissection, enzymatic digestion and straining to isolate single-cell suspensions for subsequent analysis. Typically experiments involve surface profiling of tetramer-labelled T cells (e.g. for PD-1, TIM-3, LAG-3) from PBMCs or single-cell suspensions of tissue, measurements of cytokine production (i.e. IFN-γ, TNF-α, IL-2) and proliferation assays. To stimulate isolated cells *ex vivo*, researchers pulse PBMCs with antigens over several days, and subsequently analyse tetramer^+^ T cells for functional outputs, or in the case of tissue samples, isolate T cells via flow cytometry or CD3 microbeads and then perform experiments [96]. Most studies have not examined exhaustion *per se* but rather hyporesponsive T cells exhibiting reduced cytokine production and altered surface profiles. However, more recent efforts have performed single-cell RNA-seq and mass cytometry on T cells isolated from patient tumour tissue, providing more extensive transcriptional and surface descriptions of exhausted T cells [92, 96–100].

Table 2 summarizes the approaches and caveats of current isolation/experimentation techniques in mice and human T_ex_ cells. A major caveat of these isolation approaches is that it is difficult to assign cause and effect for driving phenotypes in general. While informative of the existing state of T cells within a patient, the approaches to isolate cells from humans cannot be applied to mechanistic studies that give rise to exhaustion owing to the limited number of cells isolated from patients, and the inability to track their differentiation/activity over time *in situ*. Isolation-based approaches are also dependent on access to patient cells/tissue, which can be a barrier to experimentation.

**Table 2.** Summary of approaches and caveats of current isolation/experimentation techniques in mice and human T_ex_ cells

	T_ex_ isolated from infected/tumour model mice	T_ex_ isolated from human virally infected/tumour tissue
Method of isolation	Adoptive transfer of T cells with known antigen specificity first isolated from CD45.1 congenic mice into virally infected/tumour CD45.2 mouse models. CD45.1^+^ T cells are then re-isolated from the spleen/blood/tissue of CD45.2^+^ mice and phenotypically/functionally characterized.	Either bulk T cells or T cells of known antigen specificity (isolated using MHC tetramers, which requires prior knowledge of T-cell antigen specificity) are isolated from single-cell suspensions of mechanically digested tissue explants. T cells are typically considered exhausted based on surface markers (i.e. PD-1^+^).
Time to isolate T_ex_ cells	Up to ∼30 days after transfer of T cells into a liver tumour mouse model [84].	Obtained immediately from tissue.
	Isolation and experimentation of T_ex_ cells from virally infected mice will occur between 8 and 30 days post-infection.	
Cell yields for future testing	Low. For clone 13-based induction of exhaustion, typically, <1 × 10^6^ P14 T cells are transferred into, and re-isolated from, an infected mouse. In tumour-based models of exhaustion more cells may be transferred. For example, 2 × 10^6^ T cells of known antigen specific (OT-1 T cells that recognize OVA peptide) were used in an OVA-expressing melanoma model [85]. Owing to limited cell number, researchers may need to pool cells from several mice to perform both functional and ‘omic’-based approaches.	Limited. Very few T cells of known antigen specificity that are additionally PD-1^+^ will be obtained. This makes functional and/or ‘omic’-based characterization difficult. More recent studies will sort T cells from digested tissue directly for single-cell analysis.
Access to samples	High as many institutes with an immunology focus have access to a mouse house or mouse studies.	Low and variable. Will likely require a medical collaborator from specific hospitals to obtain tissue, and tissue samples will likely be received irregularly.
Mechanistic studies	Possible as genetically modified T cells can be adoptively transferred and tracked *in situ* through re-isolation of T cells from spleen/tissue/blood of infected mice.	Very difficult/not possible as the establishment of T_ex_ cannot be tracked *in situ* within human tissue explants. However, it may be possible to follow the establishment of human T_ex_ via transfer of T cells of known antigen specificity into patient-derived organoids.
Assigning cause–effect	Difficult to assign cause–effect as it is challenging to parse out the many signals derived from different cell–cell interactions and the microenvironment.	Not possible. Analysis of T_ex_ isolated from human tissue only provides a snapshot of their state and influence of the local microenvironment.

To overcome this, several labs have turned to *in vitro* approaches to generate large numbers of mouse and human exhausted T cells within shorter time periods (i.e. 5–14 days). Although successful in recapitulating the hyporesponsiveness of T cells as well as similarities in surface protein expression profile, reduced cytokine production and altered transcription factor activity, it remains unclear how well this captures the total and/or diverse transcriptional and epigenetic landscape of exhausted T cells *in vivo* [101–103], particularly as microenvironmental signals, and the diversity of cell–cell interactions with other immune cells [e.g. myeloid-derived suppressor cells, T_regs_, cancer-associated fibroblasts, tumour-associated macrophages (TAMs)], are known to influence T-cell exhaustion, with their specific contributions still being parsed out [46, 49, 57, 104–107]. That being said, *in vitro* approaches are easy to establish and have controllable complexity, therefore are possibly becoming the go-to approach in the future at least for large-scale and systematic dissection of key factors driving exhaustion. Below, we will discuss in detail how these new *in vitro* approaches for T-cell exhaustion are being used in the field.

### *In vitro* approaches to generate exhausted T cells (antibody-based stimulation)

Increasingly frequent use is being made of the continual activation of T cells using anti-CD3 +/− anti-CD28 (on plates or beads) to achieve a proxy exhaustion state owing to its ease and scalability. Balkhi *et al.* [108] were the first to produce exhausted T cells *in vitro* using cell-activating beads. Peripheral human CD4^+^ and CD8^+^ T cells were stimulated with anti-CD3/CD28 beads for 8 days, provoking the expression of increasing numbers of exhaustion markers, i.e. reduced cytokine production, upregulation of PD-1/LAG-3/TIM-3 and reduced killing capabilities [108]. Important caveats of this study were that there was no deep transcriptional, epigenetic or metabolic analysis of the cells relative to exhausted cells taken from *in vivo* models, making it difficult to judge how well they were able to recapitulate *in vivo* exhaustion. Furthermore, it is uncertain whether removing the beads after day 8 would have led to reversal of their reduced functional state, and therefore unclear whether the cells were truly exhausted. Extending this approach, Corselli *et al.* [109] performed a more extensive examination of the surface and transcriptional changes that occur with T cells stimulated for 14 days with anti-CD3/CD28 beads and IL-2 [109]. Changes in the expression of 38 surface proteins were followed using AbSeq, which uses multiplexed amplification reactions based on antibodies tagged with unique sequence identifiers, and targeted RNA-seq analysis of ∼400 genes. Future studies that include comparisons with *ex vivo* exhausted T cells isolated *in vivo* experiments would greatly extend this powerful approach.

Two studies have examined the metabolic changes that accompany T-cell exhaustion induced by antigen-expressing cells/anti-CD3-coated culture plates or anti-CD3/CD28 beads for persistent stimulation. The first study, by Verdhana *et al.* found that chronic T-cell stimulation induced *in vitro* invoked mitochondrial dysfunction via increased reactive oxygen species (ROS)-mediated inactivation of the mitochondrial electron transport chain. In their experiments, Verdhana *et al.* re-stimulated mouse T cells that had been pre-activated for 48 h with anti-CD3/CD28-coated plates, with either an OVA-expressing B16–F10 melanoma cell line or anti-CD3-coated plates for an additional 8 days [110]. Both approaches recapitulated classic exhaustion markers in the manner of Balkhi *et al.*, but not anergic ones, allowing a deep analysis of the role metabolism plays in exhaustion. Interestingly, in contrast to the experiments of Balkhi *et al.* with human T cells, Verdhana *et al.* found that plastic-immobilized anti-CD3 was sufficient to drive exhaustion *per se*. The reasons for these differences are unclear, but Vardhana *et al.* were able to benchmark their *in vitro*-generated, exhausted cells against LCMV clone 13 isolated T cells, showing that they exhibited similar surface marker and cytokine production changes, and similar transcriptional profiles. In the second study, Delgoffe and colleagues examined the impact of microenvironmental signals (i.e. hypoxia; 1.5% oxygen) on T-cell exhaustion during persistent stimulation of pre-activated (i.e. CD44^+^) T cells with anti-CD3/CD28 beads under hypoxic conditions for 5 days [51]. Compared with cells stimulated under normoxic conditions, they observed enhanced expression of exhaustion markers (i.e. PD-1, TIM-3, LAG-3, TOX) and reduced cytokine production, which persisted for 5 more days after removal of stimulation. Transcriptional analysis showed that their *in vitro*-produced, hypoxic, continuously stimulated cells were better mimics of terminally exhausted T cells from the B16 melanoma mouse model [33] than the cells produced under normoxic conditions, suggesting that hypoxia exacerbates of accelerates exhaustion. Like Verdhana *et al.*, Delgoffe *et al.* demonstrated mitochondrial dysfunction and increased ROS production, but went further by showing that the ROS build up was promoted by the repression of transcriptional programmes that normally control ROS production. This led to the suppression of phosphatase activity (ROS being a known inhibitor of phosphatase activity [111]), reinforcing the chronic stimulation of the T cells. Nevertheless, the authors noted that their transcriptional data were not wholly supportive of the notion that they had created ‘real’ exhausted T cells, as the *in vitro* cell-derived transcriptome did not match that of TILs from the B16 melanoma model. However, these experiments highlighted the power of *in vitro* approaches to understanding drivers of exhaustion beyond antigen exposure. Notably, Delgoffe *et al.* switched from using the B16 melanoma cell line to induce exhaustion in T cells and used anti-CD3/CD28 beads instead, arguing that this avoided the confounding effects of other signals produced by tumour cells under hypoxic stress. Future experiments can begin to elucidate the additional role, if any, of these other signals.

In more recent work, Belk *et al*. [102] developed an *in vitro* system to model chronic antigen stimulation-induced T-cell exhaustion in which T cells were first stimulated for 2 days using anti-CD3/anti-CD28-coated beads and IL-2, and then continuously for another 8 days with anti-CD3 antibody-coated beads only and IL-2 [102]. These cells produced less cytokine over time and acquired an epigenetic profile like that of terminal exhausted CD8^+^ T cells, which the authors defined as a proxy exhaustion state. Owing to the scalability of their approach, producing up to 10^8^ cells/experiment, Belk *et al*. [102] were able to perform a genome-wide knockout screen of exhaustion. They compared gRNA frequencies at the end of chronic stimulation versus those in cells at the end of an acute stimulation comprising treatment with IL-2 alone rather than anti-CD3 antibodies and IL-2. In this way, Belk *et al*. [102] were able to identify candidate regulators of this proxy-exhausted state as genes whose knockout led to better proliferation of the chronically stimulated cells. Among the ‘hits’ were chromatin modifiers including Arid1a, Smarcc1 and Smarcc2. The gene hits were then tested in mouse cancer models, which confirmed that CD8^+^ T cells lacking expression of these genes exhibited better proliferative responses to the tumours. Importantly, Arid1a-deficient CD8 T cells had open a region of chromatin around the gene encoding IFN-γ and less accessible regions around classical exhaustion markers such as *Pdcd1*, *Entpd1* (CD39) and *Tox*. While this study established a powerful new approach for identifying markers of exhaustion, several limitations/caveats should be noted. First, chronically stimulating cells with beads alone cannot, of course, wholly recapitulate the complex process by which exhaustion develops in a chronic infection or a cancer setting where multiple cell types, receptor-ligand interactions and (as discussed above) microenvironmental signals contribute to the outcome. The screen by Belk *et al*. could not identify classical surface markers of exhaustion such as PD-1, for example, presumably because their ligands were not engaged in the assay system. Second, as the exhausted state was established after chronic stimulation without resting the cells, it is unclear whether the epigenetic state of the cells induced by the gene deletions was stable or not. Lastly, stimulation with anti-CD3 antibodies only could have invoked an anergic state rather than an exhausted one, a possibility that was not examined. Notwithstanding all of this, the animal experiments confirmed that in the absence of Arid1a, CD8^+^ T cells retain proliferative and cytotoxic function *in vivo* and are better at tumour clearance.

Overall, the use of anti-CD3/CD28 antibodies to chronically stimulate T cells appears to be a viable, scalable approach to producing T cells with many exhaustion-like features. It will be necessary, however, to carefully characterize and compare the functional activity and transcriptional profiles of the cells at the end of stimulation regimes, with published *in vivo* data on exhausted and anergic T cells. It needs also to be noted that chronic stimulation of T cells can induce cell apoptosis/death, and so it is important that appropriate controls and/or approaches to remove such cells from the analysis are undertaken.

### *In vitro* (antigen/target cell-based stimulation)

To better mimic physiological T-cell activation, Zhao *et al.* [101] stimulated OT-1-specific CD8^+^ mouse T cells with antigen. They incubated the cells with OVA [257–264] peptide for 5 days along with the cytokines IL-7 and IL-15. Cells stimulated for 5 days produced less IFN-γ and IL-2, minimal TNF-α, increased expression of exhaustion surface markers and exhibited reduced killing capacity. Looking beyond functional readouts, Zhao *et al.* observed transcription factor expression and methylation patterns typical of terminally exhausted T cells, i.e. increased TOX and T-bet expression, and downregulation of TCF-1, coinciding with increased methylation [8, 25]. Furthermore, the transcriptional activity of their *in vitro* exhausted T cells matched that of T cells isolated at day 30 from LCMV clone 13 infected mice. They could also rule out the induction of anergy through comparison of transcriptional data to *in vitro* anergy models [112]. Of note, Zhao *et al.* showed that 0.1% of the CD8^+^ population purified from PBMCs comprised CD11b^+^CD11c^+^ dendritic cells, which would result in a 1:1000 ratio of target cell to T cells, which is slightly lower than that *in vivo* [113, 114]. In these experiments, therefore, there will likely be contributions from both T-cell:T-cell and DC:T-cell interactions to the exhausted state, with the former dominating. Nah and Seong [115] used a similar approach to study the role of transcription factors during T-cell exhaustion *in vitro*, expanding the method to include retrovirally induced genetic modifications through viral transduction on Day 1 of the stimulation.

Recently, Trefny *et al.* performed a targeted, pooled CRISPR screen on *in vitro* exhausted T cells to identify Snx9 as a key regulator of exhaustion. To create exhausted T cells *in vitro*, they used repeated stimulation of NY-ESO-1-specific CD8^+^ T cells (transduced with the 1G4-TCR; [116]) via an irradiated T2 tumour cell line pulsed with NY-ESO-1 antigen over 12 days (adding freshly pulsed target cells every 3 days). A suite of markers was used as validation, including increased PD-1 expression, reduced cytokine production (IFN-γ, TNF-α), less specific-killing, reduced degranulation and less proliferation [103]. Further validation came with gene set enrichment analysis, which showed that their *in vitro*-generated exhausted T cells resembled those from exhausted TILs taken from an ‘atlas’ of cancer associated T cells [117]. Importantly, their exhausted cells maintained high PD-1 expression and reduced degranulation 7 days after removing the activation stimulus, strengthening the case for having generated bona fide exhausted T cells. To perform targeted CRISPR screens, Trefny *et al.* transduced CD8^+^ T cells with a vector expressing gRNA+Cas9+mCherry alongside the 1G4-TCR, sorted for these cells, and then performed their stimulation regime, with an additional 4-h stimulation step at the end to identify exhausted T cells (based on low/no degranulation). This approach revealed that Snx9 modulates the calcium response, subsequent transcription factor activity (i.e. NFAT), metabolic activity. In the absence of Snx9, IFN-γ production by T cells was increased.

The use of peptide antigens and irradiated target cells pulsed with peptides is proving useful for generating *in vitro* exhausted T cells. This approach seems to capture the core exhaustion state, yields ∼×10 more cells than that seeded for experimentation [101] and enables downstream analysis of T cells without the need for sorting, as would occur for heterotypic live cell interactions. Of note, extensive use of anti-CD3/CD28 antibodies and peptides for pulsing cells is expensive. An alternative and cheaper approach is the use of irradiated target cells genetically modified to stably express single-chain trimers presenting peptides of interest [118]. This may also simplify the protocol of Trefny *et al.*, as there would be no need to add freshly pulsed target cells every 3 days.

## Future approaches to study T-cell exhaustion

With the advent of *in vitro* approaches for generating exhausted T cells and ease of genetically manipulating primary T cells (see Ref. [119] for a review on improvements on methods to manipulate primary T cells), the possibility of performing systematic, large-scale analyses of the genetic regulators of exhaustion is emerging. However, it will continue to be necessary to validate findings in suitable mouse models. Taking advantage of their convenience and scalability, we can expect the systematic analysis of *in vitro* exhaustion models to benefit from the ‘dialing in’ of complexity alongside maintaining persistent antigen exposure. This will include adding paracrine signals (e.g. growth factors, cytokines, metabolites), altered microenvironments, e.g. hypoxia, pH acidification, 2D versus 3D settings and extracellular matrix components, live target cells that may include genetic modifications to surface proteins/secreted proteins of interest and/or the additional cell types present in tissues alongside exhausted T cells. More advanced systems can include organoid culture systems, organ/tumour-on-a-chip and the *ex vivo* maintenance of patient-derived explants. Inevitably, with each increase in system complexity, controls will become even more important. For example, each different target cell incorporated alongside exhausted T cells will generate contact-dependent and independent signals that will need to be deconvoluted. Below, we discuss how future *in vitro* studies on T-cell exhaustion may be undertaken.

### Additional paracrine signals and cell–cell interactions

The inflammatory milieu of chronically infected tissues or tumours has an important role in regulating CD8^+^ T-cell responses [9, 120–122]. Although not sufficient to drive exhaustion alone, it very likely influences the progression, kinetics and ‘depth’ of the exhausted state. The inflammatory milieu comprises paracrine signals (e.g. cytokines, metabolites) and contact-mediated interactions (e.g. PD-1 engagement with PD-L1). An important example of the former is IL-10, which is typically elevated in chronic HBV, HCV and HIV [123], and whose blockade enhances T-cell responses during LCMV clone 13 infections [124]. The cooperation of IL-10 and T_regs_ (along with IL-35) is suggested to promote T-cell exhaustion in murine and human non-small cell lung cancer [125]. Metabolic influences on T-cell responses, typically stemming from tumour/T-cell competition and/or other immune cell activity, include glucose and amino acid (e.g. arginine and tryptophan) depletion, lactate build up, altered lipid availability and ion availability [126–130]. Many metabolic changes are yet to be directly linked to exhaustion but are worth examining given their known impact on T-cell responses. The roles of other extracellular signals thought to influence exhaustion are reviewed elsewhere [9, 129]. In the immediate future, we expect the influence of cytokines, metabolites and their combinations to be tested *in vitro* alongside anti-CD3/CD28 beads or target cell line-mediated stimulation of CD8^+^ T cells. It would, of course, be useful to measure the levels of these extracellular signals in physiological settings (or at least perform titrations) to appropriately gauge their influence, if any. Such changes may not always be relevant *in vivo* owing to the adaptability of cells, e.g. with metabolite availability [131]. Nonetheless, such screens will be useful for identifying the most important potential mediators of exhaustion.

It is now known that other tumour-associated immune cells can modulate the exhausted phenotype. For example, T_regs_, MDSCs and TAMs are commonly found in tumours and all have been implicated in helping drive T-cell exhaustion [132]. Although less well-studied, these cells are being increasingly implicated in chronic viral infections [133]. Typically, studies that characterize the impact of other immune cells on T-cell exhaustion are performed using mouse models or human tissue *ex vivo*, where it is difficult to assay their contributions to exhaustion, e.g. whether their influence is contact-dependent/independent, and to assign causality. Co-culturing these cells with CD8^+^ T cells, *ex vivo/in vitro* systems will therefore likely be helpful. As an example, Krummel and colleagues have shown that the direct interaction of CD8^+^ T cells with TAMs, one of the most abundant antigen-presenting cells in the TME [134–136], was, in part, responsible for driving exhaustion in mouse tumour models. Interestingly, the *ex vivo/in vitro* co-culture of TAMs and T cells isolated from mice revealed that the T cells formed stable interactions with TAMs and more so than with bone-marrow-derived dendritic cells, leading to weak but constitutive signalling. Building additional complexity into their system, they examined the impact of hypoxia, with the data suggesting that TAMs, especially, may prime T cells for exhaustion under hypoxic conditions. However, the only fraction of PD-1+ cells was used to support these observations, and deeper analyses are needed. Nevertheless, the value of increasingly complex *in vitro* approaches for parsing out potential drivers of exhaustion was established. One issue, however, is that the isolation and expansion of these cell types remain a significant barrier, especially from human tissue, although T_regs_ can be isolated from PBMCs, recognizing that these might not completely resemble T_regs_ from tumours, and there is considerable effort going into the development of methods for isolating TAMs and MDSCs for use *in vitro* [137–139]. Prior to performing co-culture experiments, it will be important to characterize target cells of interest to an appropriate standard as in the case, e.g. of MDSCs [140].

### 3D spheroid and organoid culture systems

Since the early 1900s, *in vitro* cellular experiments have mostly been performed in 2D [141]. While this has its merits, i.e. simplicity, accessibility, affordability and suitability for most if not all forms of microscopy, it fails to capture physiological tissue structure, e.g. the 3D shape and polarization of cells, the organization of tissue, interactions with the extracellular matrix, the diversity of cell–cell interactions and the asymmetric signals and nutrient availability characteristic of tissue [142, 143]. 3D models are therefore being developed for the analysis of cell behaviour and function, which broadly fall into two categories: spheroids and organoids. Spheroids are 3D cell aggregates generated from a single cell type, typically adherent cell lines but also cells from patients, with added complexity achievable with the incorporation of additional cell types. Spheroids often form without the need for a scaffold, being inducible using hanging-drop or suspension culture approaches. Large spheroids of several hundred micrometres in diameter tend to induce oxygen and nutrient gradients, with their protein and gene profiles often resembling *in vivo* profiles [144, 145]. As a relevant and recent example of their use to study T-cell behaviour, Ou *et al.* [146] characterized T-cell infiltration and activity in tumour spheroids derived from melanoma cell lines. They added additional complexity by incorporating cancer-associated fibroblasts into the spheroids, noting their suppressive effect on T-cell infiltration and activation. Such systems, modified to incorporate antigen specificity, could be used to induce chronic stimulation and activation of T cells in a more solid tumour-like setting. Spheroids are well suited to high-throughput experimentation allowing, e.g. cytokine/metabolite screens to identify agents that alter exhaustion progression/reversion, but this is yet to be tried. Potential caveats include the fact that spheroids are nevertheless relatively simple structures that show some variability in production [147].

Organoid cultures offer a potentially more powerful 3D *in vitro* context for studying exhaustion. Organoids are self-organizing 3D structures established from stem cells or patient-derived tissue/tumour biopsies. Tumour-derived organoids are produced simply by mechanically or enzymatically digesting tumours into single-cell suspensions that are then incorporated into collagen matrices (e.g. Matrigel) to grow. Organoids mimic the architecture and function of the origin tissue and can comprise multiple cell lineages. Most importantly, organoids can be made to retain immune cell populations, as discussed below. Caveats include the need for customized and more complex culture conditions to ensure growth of a diverse population of cells, the issue of patient heterogeneity and cost. However, we expect the use of patient-derived T cells and autologous tumour organoids to become an extremely useful setting for creating and examining *in vitro-*generated exhausted T cells. Although challenging, this is now a realistic possibility, since it has now been shown that tumour-reactive T cells can be generated by culturing PBMCs and tumours derived from the same patient in air–liquid interface or microfluidic flow systems (see, e.g. [148]), in this way allowing for the development of antigen-driven exhaustion driven by paracrine signalling and cell–cell interactions in a highly physiological microenvironment (e.g. with appropriate oxygen gradients). Caveats of this approach are that there will likely be constraints on reproducibility owing to patient heterogeneity and non-standardized culture approaches, limited total numbers of T cells for analysis, i.e. scalability (although this could be circumvented by pre-stimulating T cells from PBMCs *in vitro* to expand them before their incorporation into the organoid culture) and the lack of a stable stromal compartment associated with the TME over the course of long-term culture [149], although this may be changing [150–152]. While the method is yet to be applied to the analysis of exhaustion *per se*, single-cell RNA-seq analysis has shown that day 7 patient-derived tumour organoids do express exhaustion markers, i.e. PD-1, LAG-3 and TIM-3, underlining the potential value of this approach [150].

In addition to tumours, viral infections can be studied in organoids, opening a path to studying T-cell exhaustion in chronic viral settings (i.e. HBV, HCV and HIV). For example, *ex vivo* HBV infections and viral replication were established by infecting patient-derived healthy liver organoids with either recombinant HBV or HBV-infected patient serum or using HBV-infected patient liver explants [153]. Liver organoids were generated by isolating, expanding and differentiating primary EpCAM+ bile-duct cells derived from liver biopsies into hepatocytes *in vitro* [154, 155]. The cells could be expanded and biobanked and were amenable to genetic modification. However, access to such models will likely remain low owing to the need for patient liver biopsies and the complicated cell culture procedures required. More recently, Ott and colleagues used a commercially available microfluidic chip to co-culture liver organoids with CD8^+^ T cells specific to a HCV-derived antigen that was isolated using tetramers and expanded using irradiated PBMCs [156]. The CD8^+^ cells were responsive to HCV antigen pulsed into the liver organoid system, indicating the promise of studying chronic T-cell activation in such a setting. Human tonsil organoids infected with HIV-1 have also been used to study the metabolic impact of HIV-1 infection on the induction and expansion of dysfunctional T_regs_ [157]. Although exhaustion was not the aim of this study, altered T-cell responses upon HIV-1 infection were observed, indicating the potential of the approach for studying exhaustion. Importantly, exhaustion-like markers expressed by tissue resident CD8^+^ T cells were observed among cells in the tonsils of HIV-infected patients, supporting the use of this tissue [158].

### Organ/tumour-on-a-chip systems

Finally, ‘organ/tumour on a chip’ models are microfluidic cell culture devices that recapitulate 3D environments, tissue interfaces, vascular perfusion and nutrient and signal gradients. Such a system, recapitulating proliferating, quiescent and necrotic layers of solid tumours, was used to study exhaustion in NK cells. Ayuso *et al.* [159] placed the MCF7 breast cancer line embedded in a collagen gel into the central microchamber of the chip, with an endothelial lumen generated from an endothelial cell line, running through the collagen gel at one end, which could be used to perfuse NK cells, nutrients or other reagents. The method recapitulated tumour microenvironmental stresses, e.g. nutrient deprivation, pH changes and waste product accumulation, created as a gradient from the lumen, that enhanced ‘exhaustion-like’ features in NK cells analogous to those of T cells, e.g. upregulation of PD-1 and reduced expression of TNF-α. This example utilized a cell line. Combining the approach with patient-derived organoids could come close to mimicking solid tumours and their accompanying vasculature [160].

## Conclusion

**Figure 2.**
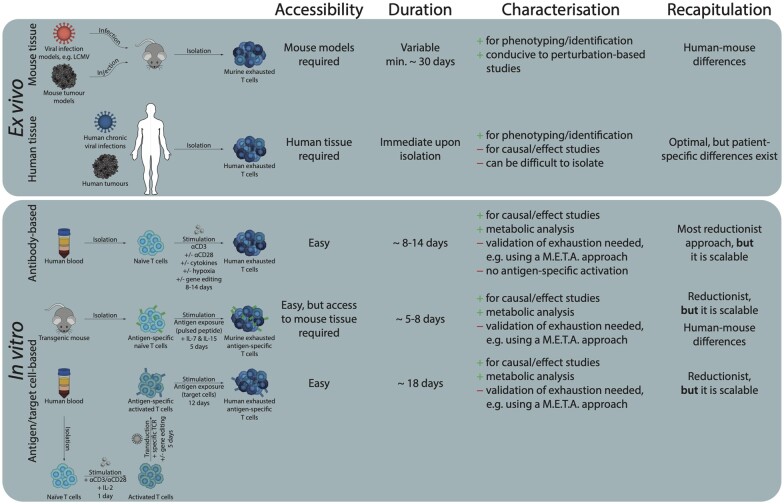
Summary of the different approaches to generate/isolate exhausted T cells. The methods can be broadly divided into *ex vivo* and *in vitro* approaches. In an *ex vivo* approach exhausted T cells are either isolated from well-established mouse models of exhaustion or from human patients whereas in an *in vitro* approach, exhausted cells are generated through different activation regiments as summarized.

Exhaustion has been studied extensively using *in vivo* mouse models and T-cells isolated from tumours and/or chronically infected tissue. Owing to limitations in these approaches—to do mostly with scalability and throughput as discussed above—the field is moving towards *in vitro* approaches which are more likely to provide detailed molecular insights into how T-cell exhaustion is established and can be reversed (in Fig. 2). As this happens, it is important to note that the specific signatures of exhaustion will likely differ depending on the details of the cellular treatments, affecting the conclusions of these studies. It has been a common practice to use a set of genes and epigenetic signatures to define exhaustion and a number of studies have compared the exhaustion signatures of chronic infection models (mainly LCMV) versus cancer models as the basis for proposing that there exists a conserved and shared core regulatory network driving exhaustion [90, 95, 161]. *In vitro*-generated exhausted cells exhibit many of these key hallmarks of exhaustion, but it will be important to thoroughly characterize how well any given *in vitro* model captures the necessary phenotype. We recommend creating a ‘M.E.T.A’, i.e. metabolic, epigenetic, transcriptional and activation-based set of phenotypic markers for use in capturing the surface profiles, and cytokine production and killing activities of exhausted T cells generated *in vitro.* These four sets of markers comprise overlapping measures of exhaustion so that if at least two of these criteria are met, there will likely be good agreement that truly exhausted-state T cells are being studied. We note that as our manuscript was under review, an approach to generate exhausted mouse T cells through stimulation with live antigen-pulsed dendritic cells *in vitro* over 7 days was established by Wu et al. [162]. This preprint serves as an exemplar case for our M.E.T.A analysis as they examined three out of four markers (i.e. ETA) in their T cells at the endpoint of their stimulation regime. Given the physiological relevance of their *in vitro* approach, they then went on to identify mechanistic insights into regulators of T-cell exhaustion via a CRISPR screen [162].

Overall, with consistent and agreed protocols, it should be possible to perform experiments that are comparable across laboratories, particularly as the field moves towards more complicated systems incorporating more environmental factors in the study of T-cell exhaustion.
